# Supplementary material for: The causal relationship between the human gut microbiota and pyogenic arthritis: a Mendelian randomization study
Source: Front Cell Infect Microbiol. 2024 Nov 26;14:1452480. doi: 10.3389/fcimb.2024.1452480 (PMC11629706; doi:10.3389/fcimb.2024.1452480)
Supplement: Supplementary file 1 [file DataSheet1.zip › mendelian test/ebi-a-GCST90027801.csv_forest.pdf]

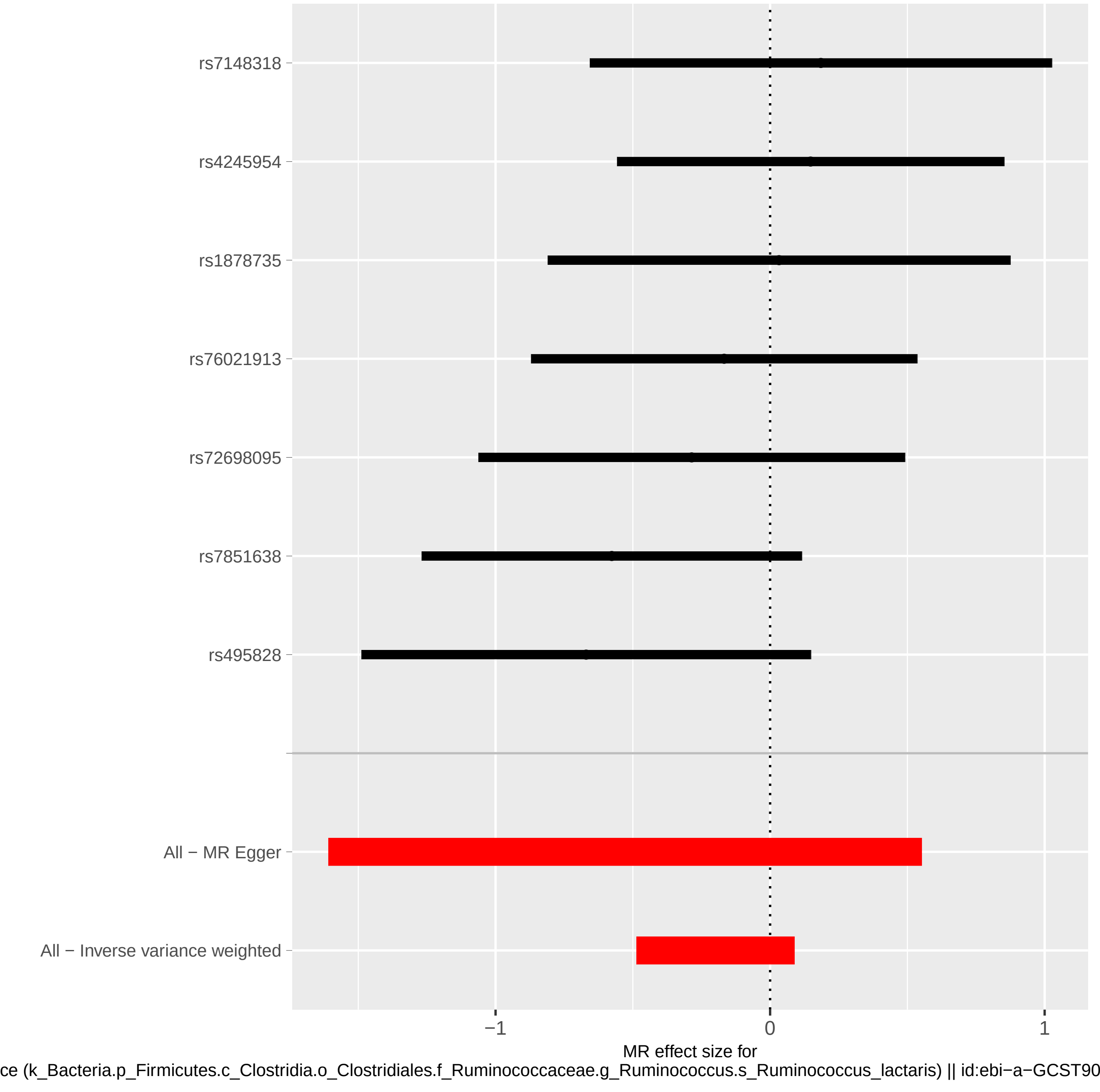
rs7148318
rs4245954
rs1878735
rs76021913
rs72698095
rs7851638
rs495828
All − MR Egger
All − Inverse variance weighted
−1
0
1
MR effect size for
ce (k_Bacteria.p_Firmicutes.c_Clostridia.o_Clostridiales.f_Ruminococcaceae.g_Ruminococcus.s_Ruminococcus_lactaris) || id:ebi−a−GCST90
